# Editorials

**Published:** 1897-12

**Authors:** 


### HOMŒOPATHIC PHYSICIAN,

A MONTHLY JOURNAL OF

HOMŒOPATHIC MATERIA MEDICA AND CLINICAL MEDICINE.

"If our school ever gives up the strict inductive method of Hahnemann, we are lost, and deserve only to be mentioned as a caricature in the history of medicine."—CONSTANTINE HERING.

Vol. XVII. DECEMBER, 1897. No. 12.

### EDITORIALS.

INOCULATION FOR DRUNKENNESS is the latest extreme that these new methods of medication have reached. To accomplish this result a horse is given whiskey, or some other form of alcohol until he is intoxicated. In this condition the poor creature is kept for six months at a time. His blood is then drawn and used to inoculate drunkards in order to cure them of their "disease." A case is reported of a child about four years old, who, from his earliest infancy, was taught to drink whiskey, and had become a confirmed drunkard, constantly demanding "Wicky." Inoculation with this new "serum" was practiced upon him, and his terrible appetite, it is now claimed, has been eradicated.

In the face of this new development of the practice of inoculation the articles on vaccination that have appeared in this journal for the last twelve months acquire a new significance. As the intent of this journal is *not* to servilely follow what may be called "public opinion" in the profession and cater to it, for the purpose of enlarging the subscription list, but rather to *lead* it into proper channels of correct thought, and to educate it, we feel that we must continue the publication for some time longer of these exposures of vaccination, notwithstanding that one or two friends of the journal have made protests more or less forcible against the course we are pursuing.

The extremes of inoculation practice demand that the earliest and most universal form of inoculation shall be made thoroughly well known in all its details that a proper resistance may be aroused against the compulsory infliction upon the community of these later and far more dangerous methods which are being announced every few weeks. In this policy we hope to be sustained by all in the profession, even those who continue to be warm, if mistaken friends of vaccination.

---

Apology.—The Editor regrets that he must once more delay the editorials on the remedies, as promised. The work of preparing the journal, attending to a considerable practice, and the accumulations of a large correspondence, altogether make an accumulation of tasks that seems almost insurmountable, and therefore some portion must be neglected. Hence the editorials fail to appear as usual.

---

A Matter of Choice.—The utter want of appreciation of the strictly scientific character of the homœopathic principle of medication, and of the responsibility incurred in assuming to practice it by certain physicians of so-called "liberal" views, is well illustrated by a story found in *The Homœopathic World*, of London, which relates that a patient having failed to recover under the old school treatment, the friends decided to put the case into the hands of a homœopathic physician, and so announced to the medical attendant, whereupon he exclaimed: "Oh, *I* can treat him homœopathically; *which medicine would you like me to give?*"

The mental state of such physicians would be amusing if it were not so contemptible.

